# The Potential of Human Pulmonary Mesenchymal Stem Cells as Vectors for Radiosensitizing Metallic Nanoparticles: An In Vitro Study †

**DOI:** 10.3390/cancers16183239

**Published:** 2024-09-23

**Authors:** Angélique Arcambal, Axelle Septembre-Malaterre, Sabrina Pesnel, Anne-Laure Morel, Philippe Gasque, Mickael Begue, Youssef Slama

**Affiliations:** 1 Laboratoire Interdisciplinaire de Recherche en Santé (LIRS), RunResearch, Sainte-Clotilde Clinic, 127 Route de Bois de Nèfles, 97400 Saint-Denis, Reunion Island, France; mickael.begue@clinifutur.net; 2 Unité de Recherche Etudes Pharmaco-Immunologiques (EPI), University of La Réunion, CHU of La Réunion, Felix Guyon Site, Allée des Topazes, SC11021, 97400 Saint-Denis, Reunion Island, France; axelle.malaterre-septembre@univ-reunion.fr (A.S.-M.);; 3 Torskal Nanosciences, 2 Rue Maxime Rivière, 97490 Sainte-Clotilde, Reunion Island, France; s.pesnel@aaz-formation.com (S.P.); annelaure.morel@torskal.com (A.-L.M.); 4 Department of Radiotherapy, Sainte-Clotilde Clinic, Clinifutur Group, 127 Route de Bois de Nèfles, 97400 Saint-Denis, Reunion Island, France

**Keywords:** mesenchymal stem cells, Fe_3_O_4_@Au nanoparticles, oxidative stress, proinflammatory response, protumorigenic markers, vectorization, innovative therapies, radiotherapy, radiosensitizers, lung cancer

## Abstract

**Simple Summary:**

Currently, delivering nanoparticles to hard-to-reach tumor sites remains a challenge in nanomedicine. Mesenchymal stem cells are an innovative strategy for targeting tumors, and genetic engineering could enable them to release antitumor agents and/or nanomaterials at tumor sites. Therefore, combining radiosensitizing agents with mesenchymal stem cells represents a promising strategy in the fight against cancer. Still, evaluating whether nanoparticles can modulate mesenchymal stem cells’ behavior is essential. This study assessed the impact of new Fe_3_O_4_@Au nanoparticles on human pulmonary mesenchymal stem cells to determine whether they can be used as carriers for radiosensitizer agents to cancer sites. This study focused on the markers related to cell death, redox and proinflammatory status, and tumorigenesis.

**Abstract:**

Background/Objectives: Metallic nanoparticles (NPs) exhibit interesting radiosensitizing effects, and finding a way to accurately deliver them appears to be crucial. Due to their tumor tropism, mesenchymal stem cells (MSCs) represent a strategic approach. Therefore, we aimed to evaluate the impact of core–shell Fe_3_O_4_@Au NPs on the functionality of human pulmonary MSCs (HPMSCs). Methods/Results: The results showed that 100 µg/mL Fe_3_O_4_@Au NPs, accumulated in HPMSCs (revealed by Prussian blue staining), did not alter cell viability as assessed by cell counting, MTT, and LDH assays. However, *caspase 9* and *Bcl2* gene expression, evaluated by RT-qPCR, was regulated 72 h after exposure to the NPs. Moreover, the NPs also decreased proinflammatory cytokine/chemokine secretions, except for CXCL8 (ELISA). These modulations were associated with the downregulation of *AMPK* gene expression at 24 h. In contrast, the NPs did not modulate *VEGF*, *PI3K*, or *PDGF* gene expression. Nevertheless, a decrease in VEGF secretion was observed after 24 h of exposure to the NPs. Interestingly, the Fe_3_O_4_@Au NPs did not modulate *Nrf2* gene expression, but they did regulate the expression of the genes encoding Nox4 and HMOX-1. Additionally, the NPs increased ROS production, suggesting a redox imbalance. Conclusions: Finally, the Fe_3_O_4_@Au NPs did not affect the HPMSCs’ viability or proangiogenic/tumorigenic markers. These findings are encouraging for investigating the effects of Fe_3_O_4_@Au NPs delivered by HPMSCs to tumor sites in combination with radiation.

## 1. Introduction

Cancer is one of the most significant public health challenges. The Globocan report, published in 2024, estimated that there were 20 million new cases of cancer and 9.7 million deaths in 2022 [1]. Among the proposed therapies for cancer management, radiotherapy (RT), for both curative and palliative treatment, represents one of the most efficient non-surgical therapies and is utilized in 50% of cases [2]. RT is based on ionizing radiation, which targets and destroys cancer cells. High-energy ionizing radiation can directly and indirectly cause DNA damage in tumor cells, leading to cell cycle arrest, alterations in cell proliferation, and, ultimately, cancer cell death through apoptosis and necrosis [3,4]. RT strikes DNA, causing DNA breaks, and increases reactive oxygen species (ROS) levels through water radiolysis, which stimulates the production of endogenous secondary ROS, ultimately leading to oxidative stress and the death of cancerous cells.

Notably, RT-induced oxidative stress is still associated with a proinflammatory response, implicating the release of cytokines, such as tumor necrosis factor-α (TNF-α), interleukin (IL)-1β, and IL-6, and chemokines, such as CXCL8, which participate in immune cell recruitment [5,6].

While RT is a critical and effective tool in the fight against cancer, it also has limitations that require the development of innovative strategies to overcome them. Particle accelerators, imaging tools, and related software have continuously advanced to enhance its precision, minimize its toxicity to healthy tissue, and maintain radiation effectiveness against tumor cells. To achieve maximum effectiveness and an optimal related cure rate, the radiation dose must be delivered with exceptional precision to the tumor while sparing the surrounding healthy tissues and the organs at risk. A low radiation dose may be associated with a risk of cancer recurrence, whereas a high radiation dose increases the risk of chronic and late toxicities [7,8,9,10].

Non-small-cell lung cancer (NSCLC) is still the leading cause of cancer-related deaths in the world, and its management during different stages includes surgery, chemotherapy, and radiotherapy. Depending on the cancer’s stage, surgery can be replaced by brachytherapy, photodynamic therapy, or laser therapy [11]. However, stages IVA and B of NSCLC can be challenging to cure, demonstrating the importance of developing early diagnostic tools. Advanced NSCLCs are known to become resistant to current treatments, and radioresistant cancers necessitate increased radiation doses for tumor control, leading to a heightened risk of developing radiation-induced cancer [7]. In NSCLCs, increasing the radiation dose improves overall survival but also increases the risk of pulmonary and cardiac toxicity [12].

In this context, there is a genuine interest in developing new therapeutic strategies, such as radiosensitizers, that could enhance the effects of ionizing radiation on tumors without increasing the administered dose.

Due to the high surface–volume ratio of metallic nanoparticles (NPs), we observed major hepatosplenic uptake that could be monitored by chemical functionalization, which could induce higher stability in physiological conditions. The high surface–volume ratio of NPs also involves superior electrical and optical attributes correlated with specific catalytic capabilities [13]. Moreover, the literature data have reported that a glucose coating increases the kinetics of internalization in tumor cells [14].

Once accumulated in cancerous cells and combined with ionizing radiation, NPs demonstrate a notable radiosensitizing effect, which may vary depending on the specificity of the studied cells [15,16]. Ionizing radiation interacts with NP and, more precisely, with atoms with high atomic numbers (Z), generating secondary photoelectrons that primarily promote ROS production [3,17,18]. Indeed, gold, silver, or iron NPs potentiate the therapeutic effect of ionizing radiation by amplifying redox imbalance, mitochondrial dysfunction, the alteration of the cell cycle, and defects in double-strand break (DSB) repair, as well as affecting cell proliferation [19,20].

Metallic NPs can also increase the apoptosis and necrosis of tumor cells, decrease cancer cell invasion and clone formation, as well as contribute to the inhibition of primary tumors and metastasis, either stimulated by light-based therapies and near-infrared laser or coupled with radiotherapy or therapeutic agents, such as siRNA, drugs, or biomolecules [21,22,23,24]. Furthermore, NPs can be used for diagnostic purposes and, in combination with other therapeutic approaches and tools, such as light-based therapies, exhibit anticancer applications with specific features, such as spatiotemporal control, minimal invasiveness, or high selectivity [25,26].

However, the challenge of utilizing nanomedicine as a therapeutic tool lies in effectively delivering nanoparticles to cancerous sites, particularly those that are challenging to access intratumorally.

Over the past two decades, there has been a growing interest in mesenchymal stem cells (MSCs) and their therapeutic potential. MSCs exhibit anti-inflammatory, antioxidant, and immunoregulatory capacities, which are relevant for applications in regenerative medicine, as well as a tropism toward tumor sites, which seems strategic for developing new anticancer therapies [27,28].

Importantly, it has been demonstrated that MSCs naturally accumulate in the lungs 24 h after their systemic administration in mice, rabbits, and monkeys, showing that MSCs could be particularly indicated for delivering therapeutic agents to tumors in the case of NSCLC [29,30]. Kidd et al. also showed the specific and persistent accumulation of intraperitoneal and systemic administrated MSCs in inflammatory and tumor sites [31].

Consequently, advancements in genetic engineering techniques have enabled MSCs to regulate the release of antitumor agents at the tumor site [32,33]. Data from the literature have also reported that light-based therapy coupled with nanoparticles can trigger mesenchymal stem cell delivery for therapy and imaging [26].

Combining radiosensitizing agents with MSCs has recently shown promising antitumor properties. Indeed, adipose-derived MSCs carrying bismuth selenide NPs administered intravenously significantly accumulated in the lung tissues of orthotopic A549 tumor-bearing mice, and NPs combined with X-ray irradiation improved the survival rate of mice [29]. However, studies also demonstrated that MSCs recruited at tumor sites can promote tumor growth through exosome release [28,34]. There is still a lack of data regarding the biological impact of nanoparticles on mesenchymal stem cells (MSCs) used as carriers during cancer treatments.

In this context, it appeared crucial to first evaluate the response of MSCs to therapeutic agents, such as metallic nanoparticles, used as radiosensitizers and to choose optimal conditions with which to validate MSCs as promising vehicles for nanomedicine during lung cancer, as well as ensure they are reliable and do not contribute to cancer progression.

Thus, this study aims to assess the impact of novel green core–shell Fe_3_O_4_@Au nanoparticles on the functionality, inflammatory response, and redox status of human pulmonary MSCs (HPMSCs) to underscore their potential as carriers for radiosensitizer agents at the cancerous site. The tested NPs are hybrid particles that combine ferromagnetic Fe_3_O_4_ NPs coated by gold (Au) atoms to leverage their radiosensitizing effects while minimizing toxicity risks. Moreover, NPs represent an interesting strategy due to their eco-friendly synthesis process using a sonochemical approach.

Various markers related to redox status, proinflammatory responses, protumorigenic effects, and cell death were examined.

## 2. Materials and Methods

### 2.1. Synthesis of Fe_3_O_4_@Au Nanoparticles

Fe_3_O_4_@Au NP synthesis was established in patent FR3131850A1 by “Morel Anne-Laure, Ben Haddada Maroua, Nikitenki Serguei, Chave Tony, 2022, synthèse assistée par ultrasons de nanoparticules constituées d’un cœur ferrique recouvert d’or” [35] and performed in the present study, according to a previously published method [19], using a sonochemical approach to follow the principles of green chemistry. Details of the synthesis and characterization of Fe_3_O_4_@Au nanoparticles studied in the present study have been described by Slama et al. [19].

### 2.2. Cell Culture

Primary human pulmonary mesenchymal stem cells (HPMSCs) isolated from human lung tissue were purchased from the ScienCell research laboratory (Carlsbad, CA, USA) and were cultured in Modified Eagle’s Medium (MEM; PAN Biotech, Aidenbach, Germany) supplemented with 10% heat-inactivated fetal bovine serum (FBS; PAN Biotech), 1 mM of sodium pyruvate (PAN Biotech), 0.5 µg/mL of amphotericin B (PAN Biotech), 2 mM of L-glutamine (Biochrom AG, Berlin, Germany), and 0.1 mg/mL penicillin–streptomycin (PAN Biotech). Then, cells were placed in a humidified 5% CO_2_ incubator at 37 °C.

### 2.3. Effect of Fe_3_O_4_@Au Nanoparticles on the Viability of HPMSCs

HPMSCs were cultured in a 96-well plate (5000 cells/well), where they grew for 24 h at 37 °C/5% CO_2_. Next, HPMSCs were exposed to increasing Fe_3_O_4_@Au NP concentrations (10 to 500 μg/mL) for 24, 48, and 72 h. Mitochondrial metabolic activity was measured by a 3-(4-5-dimethylthiazol-2-yl)-2,5-diphenyltetrazolium bromide (MTT) assay. After 24, 48, and 72 h of exposure to NPs, culture media were replaced by a mix of 20 µL of sterile-filtered MTT solution (5 mg/mL, Sigma-Aldrich, St-Louis, MO, USA) and 200 µL of culture media and were incubated at 37 °C for 2 h. Then, the plates were centrifuged, and the media were removed. Dimethyl sulfoxide was used to dissolve formazan crystals. Optic density (OD) was obtained at 550 and 670 nm (Cytation 5, BioTek, Agilent, Santa Clara, CA, USA).

The data are presented as a percentage of viable/active cells compared to the control conditions (100% of viable cells):

Mitochondrial metabolic activity (% of viable/active cells) = (OD _treated cells_/OD _control condition_) × 100

In parallel, cell counting was also performed using the trypan blue exclusion method after 24, 48, and 72 h of exposure to Fe_3_O_4_@Au NPs:

Cell counting (% of viable cells) = (Cell count _treated cells_/Cell count _control cells_) × 100

### 2.4. Measurement of the Cytotoxic Effect of Fe_3_O_4_@Au Nanoparticles

The cytotoxicity of NPs was determined using a non-radioactive cytotoxicity assay kit (Promega, Madison, WI, US), measuring the level of lactate dehydrogenase (LDH) released from damaged cells. Cells grew in a 96-well plate (5000 cells/well) and were incubated with 100 µg/mL Fe_3_O_4_@Au NPs. Control cells were lysed as indicated by the manufacturer to obtain the maximum LDH release, and the supernatants were collected 24 or 72 h after exposure to NPs. The OD was read on a spectrophotometer at 490 nm (Cytation 5, BioTek, Agilent).

The percentage of cytotoxicity was expressed relative to the maximum LDH release induced by the addition of Triton-X100 1% (Sigma-Aldrich) and determined via the following calculation:

% cytotoxicity = (Experimental LDH release/Maximum LDH release) × 100.

Control refers to LDH release from cells not exposed to nanoparticles.

### 2.5. Cellular Uptake of Fe_3_O_4_@Au Nanoparticles

The uptake of Fe_3_O_4_@Au NPs by HPMSCs was determined using a Prussian blue assay. Cells were cultured for 24 h in a 6-well plate (70,000 cells/well) at 37 °C/5% CO_2_. Then, HPMSCs were incubated with 100 µg/mL Fe_3_O_4_@Au nanoparticles for 24 or 72 h. In accordance with Khoei et al. [36], cells were stained using the Prussian blue method, and the uptake was evaluated under light microscopy (Nikon microscope, Amstelveen, The Netherlands).

### 2.6. Measurement of the Expression of Genes by RT-qPCR Analysis (SYBR Green)

HPMSCs were placed in 6-well plates (70,000 cells/well) and allowed to grow for 24 h. Then, cells were incubated with 100 µg/mL Fe_3_O_4_@Au NPs for 24 or 72 h before the total RNA extraction (Quick-RNA™ Viral Kit, Zymo Research, Freiburg, Germany); RT-qPCR was performed using a Bioline Sensifast Probe NO-ROX One Step Kit (Meridian Bioscience, Cincinnati, OH, USA) supplemented with the SYBR Green reagent (Lonza, Visp, Switzerland). Following reverse transcription, qPCR was carried out with a Quantstudio 5 PCR thermocycler (Thermo Fisher Scientific, Waltham, MA, USA). The GAPDH was used as a housekeeping reference gene. All of the primer sequences related to the studied genes are listed in Table 1.

### 2.7. Measurement of ROS Generation

Intracellular ROS levels were determined using the non-fluorescent cell-permeant probe 2′,7′-dichlorodihydrofluorescein diacetate (DCFH-DA, Sigma-Aldrich), which is converted into highly fluorescent 2′,7′-dichlorofluorescein (DCF) after being oxidized by intracellular ROS. The HPMSCs were seeded in 96-well black plates (5000 cells/well) for 24 h, before being incubated with 100 µg/mL Fe_3_O_4_@Au NP for 2 h. Then, cells were washed with PBS twice and incubated with the DCFH-DA probe (10 µM) for 45 min. Then, DCF fluorescence was quantified at an excitation wavelength of 485 nm and an emission wavelength of 530 nm (Cytation 5, BioTek, Agilent).

The data are presented as a percentage of intracellular ROS level compared to the control conditions (100% intracellular ROS level):

Intracellular ROS levels (%) = (DCF fluorescence _treated cells_/DCF fluorescence _control cells_) × 100

### 2.8. Quantification of Released Cytokines, Chemokines, and Growth Factor 

HPMSCs were cultured in 6-well plates (70,000 cells/well) for 24 h before being incubated with 100 µg/mL Fe_3_O_4_@Au NPs for 24 and 72 h. Then, supernatants were collected, centrifuged, and analyzed using human ELISA kits to quantify the release of TNF-α, IL-1β, IL-6, CXCL8, CCL5, and VEGF (PeproTech, Cranbury, NJ, USA) according to the manufacturer’s recommendations. The absorbance was read at 450–570 nm (Cytation 5, BioTek, Agilent).

The cytokine, chemokine, and growth factor secretions were determined using the slope coefficient obtained from the corresponding standard curve:

Final concentration (pg/mL) = (OD_sample_/slope coefficient) × dilution factor

### 2.9. Statistical Analysis

The results are expressed as the means ± standard errors of the mean (SEMs) of n = 3 independent cellular passages. An unpaired Student’s test (*t* test) or a one-way analysis of variance (ANOVA), followed by Sidak, Tukey, or Dunnett tests, when appropriate, was performed. Statistical analysis was performed and graphics were created with the GraphPad Prism 8 program (GraphPad Prism 8.0.1 Software, Inc.). A *p*-value ≤ 0.05 was considered statistically significant.

## 3. Results

### 3.1. Effect of Fe_3_O_4_@Au Nanoparticles on the Cell Viability of HPMSCs

**Figure 1:**
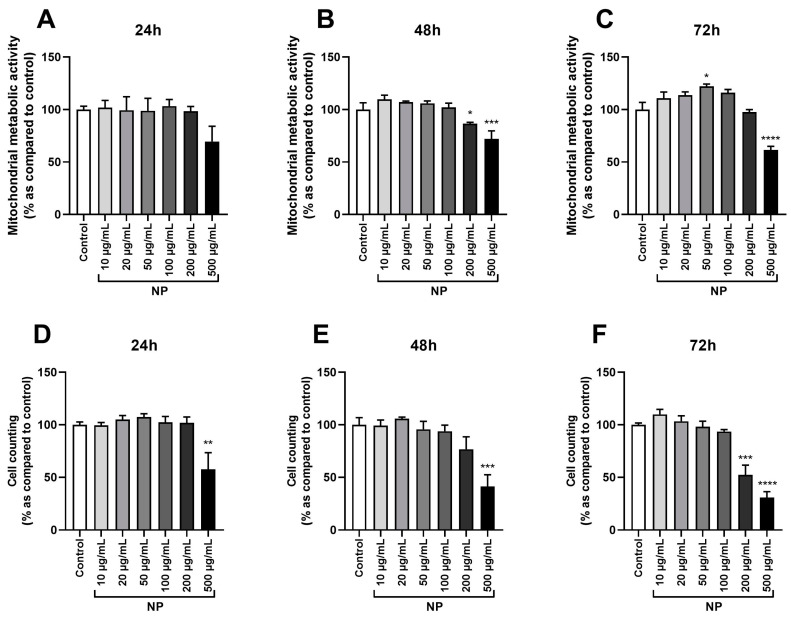
Dose- and time-dependent effects of Fe_3_O_4_@Au nanoparticles on the viability of HPMSCs. Cells were incubated with increasing concentrations of Fe_3_O_4_@Au NPs (10–500 µg/mL). (**A**) The mitochondrial metabolic activity of HPMSCs was quantified via an MTT assay after 24 h, (**B**) 48 h, and (**C**) 72 h of incubation with Fe_3_O_4_@Au NPs, and (**D**) cell counting was performed via the trypan blue exclusion method on cells after 24 h, (**E**) 48 h, or (**F**) 72 h of incubation with Fe_3_O_4_@Au NPs. Data are expressed as the mean ± SEM of three independent experiments. *: *p* < 0.05, **: *p* < 0.01, ***: *p* < 0.001, and ****: *p* < 0.0001 as compared to control conditions.

To evaluate the dose and time effects of Fe_3_O_4_@Au NPs on HPMSCs’ viability and identify the optimal work concentration for future experiments, the mitochondrial metabolic activity of cells was determined, as was cell death via cell counting. Nanoparticles did not modulate mitochondrial metabolic activity after 24 h but significantly decreased it on cells exposed to 500 µg/mL of Fe_3_O_4_@Au NPs after 48 h and 72 h of exposure to NPs (Figure 1A–C).

Cells incubated with 200 µg/mL Fe_3_O_4_@Au NPs were affected after 48 h of exposure (Figure 1B). An increase in mitochondrial activity was also noted at a concentration of 50 µg/mL after 72 h of exposure (Figure 1C). Concordant results were observed in the cell counting analysis. Fe_3_O_4_@Au NPs decreased cell viability at 500 µg/mL after 24, 48, and 72 h (Figure 1D–F) and exhibited a cytotoxic effect at 200 µg/mL after 72 h of exposure to NPs (Figure 1F). None of the lower concentrations of Fe_3_O_4_@Au NPs tested (10–100 µg/mL) affected cell viability or mitochondrial metabolic activity.

Based on these findings, the concentration of 100 µg/mL of Fe_3_O_4_@Au NP was chosen to investigate their impact on HPMSCs further. For the remainder of this study, exposure times of 24 and 72 h to NPs were selected to simulate an administration that could be practiced in clinical routine.

### 3.2. Uptake of Fe_3_O_4_@Au Nanoparticles by HPMSCs

To assess the potential of HPMSCs as carriers for NP delivery, the capacity of NPs to cross the membrane and accumulate in the cytoplasm and nucleus of HPMSCs was investigated. Prussian blue staining was conducted on cells incubated with 100 µg/mL of NPs for 24 and 72 h after multiple washes to eliminate excess NPs.

**Figure 2:**
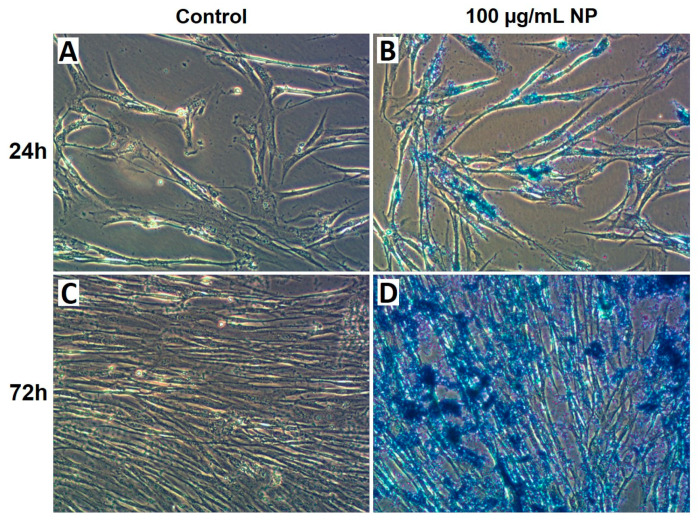
Evaluation of the cellular uptake of Fe_3_O_4_@Au nanoparticles by HPMSCs. Cellular internalization was assessed using Prussian blue staining. The internalization of Fe_3_O_4_@Au NPs was determined on (**A**) control cells and (**B**) cells exposed to 100 µg/mL Fe_3_O_4_@Au NPs for 24 h, as well as (**C**) control cells and (**D**) cells exposed to 100 µg/mL Fe_3_O_4_@Au for 72 h. Blue staining is consistent with the intracellular uptake of Fe_3_O_4_@Au NPs by HPMSCs.

The Fe (III) contained in Fe_3_O_4_ reacts with Prussian blue. Images of the staining were captured using optical microscopy and are presented in Figure 2. The blue staining demonstrated the intracellular accumulation of Fe_3_O_4_@Au NPs compared to untreated cells. After 24 h of exposure to 100 µg/mL of NPs (Figure 2B), the blue staining increased compared to the control conditions (Figure 2A). Similarly, after 72 h of exposure to 100 µg/mL of NPs (Figure 2D), the blue staining increased compared to the corresponding control conditions (Figure 2C), demonstrating the uptake of NPs.

### 3.3. Effect of Fe_3_O_4_@Au Nanoparticles on HPMSC Apoptosis and Necrosis

**Figure 3:**
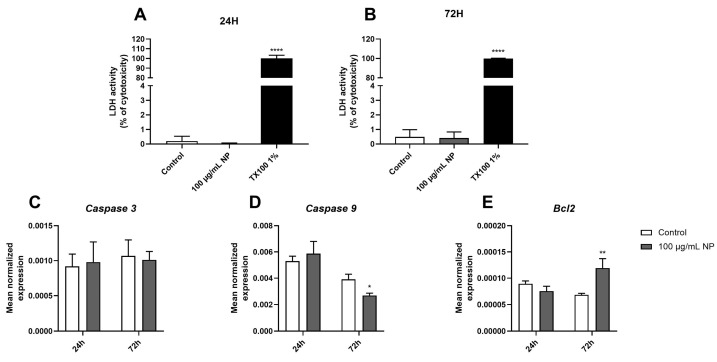
Effect of Fe_3_O_4_@Au nanoparticles on the necrosis and apoptosis markers of HPMSCs. (**A**) The cytotoxic effect of 100 µg/mL Fe_3_O_4_@Au NPs on cells was assessed using an LDH assay after 24 h and (**B**) 72 h of exposure to nanoparticles. The mean expression of genes encoding (**C**) caspase 3, (**D**) caspase 9, and (**E**) Bcl2 was obtained via RT-qPCR on HPMSCs incubated or not with Fe_3_O_4_@Au NPs for 24 h and 72 h. Data are indicated as the mean ± SEM of three independent experiments. *: *p* < 0.05, **: *p* < 0.01, and ****: *p* < 0.0001 as compared to the corresponding control conditions.

To evaluate the necrosis and regulation of apoptosis markers 24 and 72 h following exposure to 100 µg/mL of NPs, lactate dehydrogenase (LDH) activity (Figure 3A,B) and the expression of genes encoding apoptosis markers, namely *caspase 3* (Figure 3C), *caspase 9* (Figure 3D), and *Bcl2* (Figure 3E), were quantified. The results indicated that exposure to 100 µg/mL of Fe_3_O_4_@Au NPs for 24 h did not modulate the expression of apoptotic markers. However, 72 h of exposure to NPs induced a downregulation in *caspase 9* gene expression (Figure 3D) while upregulating the expression of the gene coding for Bcl2 (Figure 3E). The same results were observed for the activity of LDH, which was unchanged in the cells exposed to 100 µg/mL of Fe_3_O_4_@Au NPs for 24 h (Figure 3A) or 72 h (Figure 3B), compared to the respective control conditions. Nanoparticles at a 100 µg/mL concentration did not promote cell death by apoptosis or necrosis at the exposure times studied.

### 3.4. Effect of Fe_3_O_4_@Au Nanoparticles on the Redox Status of HPMSCs

**Figure 4:**
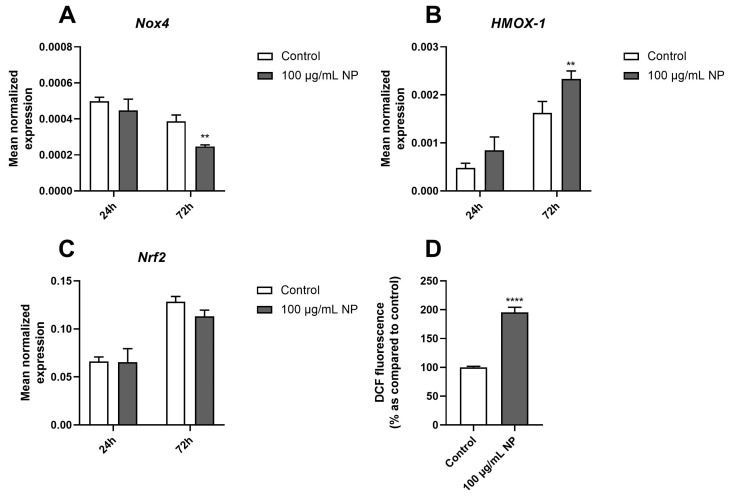
Effect of Fe_3_O_4_@Au nanoparticles on the redox status of HPMSCs. The mean normalized expression of genes encoding redox markers was determined by RT-qPCR. The expressions of genes coding for (**A**) Nox4, (**B**) HMOX-1, and (**C**) Nrf2 in HPMSCs were measured after 24 h and 72 h of exposure to 100 µg/mL Fe_3_O_4_@Au NPs. (**D**) ROS production was determined in HPMSCs exposed to 100 µg/mL Fe_3_O_4_@Au NPs for 2 h by using the DCFH-DA probe. Data are expressed as the mean ± SEM of three independent experiments. **: *p* < 0.01 and ****: *p* < 0.0001 as compared to the corresponding control conditions.

Next, we evaluated the regulation of genes encoding redox markers, such as ROS-producing enzymes, namely NADPH oxidase 4 (Nox4); redox-dependent transcription factors, namely nuclear factor erythroid 2–related factor 2 (Nrf2); and antioxidant enzymes, namely heme oxygenase-1 (HMOX-1). The results showed that 100 µg/mL of Fe_3_O_4_@Au NPs for 72 h downregulated *Nox4* gene expression (Figure 4A) while upregulating *HMOX-1* gene expression in HPMSCs (Figure 4B), but it did not modulate *Nrf2* gene expression over time (Figure 4C). Noticeably, the intracellular ROS level of HPMSCs measured using the DCFH-DA probe was increased following 2 h of incubation with Fe_3_O_4_@Au NPs compared to the control condition (Figure 4D).

### 3.5. Effect of Fe_3_O_4_@Au Nanoparticles on the Proinflammatory Response of HPMSCs

**Figure 5:**
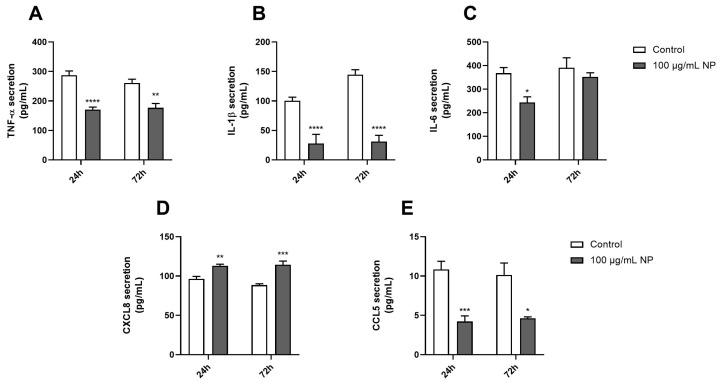
Effect of Fe_3_O_4_@Au nanoparticles on proinflammatory cytokines and chemokines secreted by HPMSCs. The proinflammatory response of HPMSCs was evaluated through ELISA. The levels of (**A**) TNF-α, (**B**) IL-1β, (**C**) IL-6, (**D**) CXCL8, and (**E**) CCL5 secreted by HPMSCs were quantified after 24 or 72 h of exposure to 100 µg/mL of Fe_3_O_4_@Au NPs. Data are presented as the mean ± SEM of three independent experiments. *: *p* < 0.05, **: *p* < 0.01, ***: *p* < 0.001, and ****: *p* < 0.0001 as compared to the corresponding control condition.

The effect of Fe_3_O_4_@Au NPs on proinflammatory status was determined by measuring the levels of proinflammatory cytokines and chemokines released by HPMSCs over time using ELISA kits. As shown in Figure 5, 100 μg/mL of Fe_3_O_4_@Au NPs for 24 h significantly reduced the secretion of TNF-α (Figure 5A), IL-1β (Figure 5B), IL-6 (Figure 5C), and CCL5 (Figure 5E) compared to the control. Notably, 72 h of exposure to NPs resulted in a similar effect as that observed at 24 h, explicitly decreasing the release of cytokines, except for IL-6, which remained identical to the control condition (Figure 5C). Importantly, only the secretion of CXCL8 (Figure 5D) was upregulated in cells exposed to NPs over time.

### 3.6. Effect of Fe_3_O_4_@Au Nanoparticles on the Expression and Production of Protumorigenic Markers on HPMSCs

**Figure 6:**
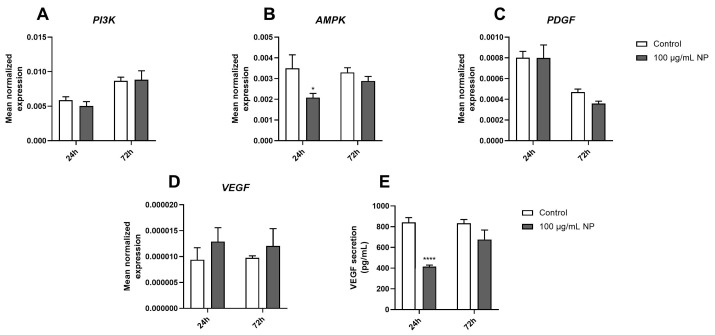
Effect of Fe_3_O_4_@Au nanoparticles on the expression and production of protumorigenic factors by the HPMSCs. The gene expression and secreted levels of protumorigenic markers were measured on cells exposed to 100 µg/mL Fe_3_O_4_@Au nanoparticles for 24 and 72 h. First, the mean expression of genes encoding (**A**) PI3K, (**B**) AMPK, (**C**) PDGF, and (**D**) VEGF were determined via the use of RT-qPCR. Then, (**E**) the level of VEGF secreted by HPMSCs was quantified through an ELISA. Data are expressed as the mean ± SEM of three independent experiments. *: *p* < 0.05 and ****: *p* < 0.0001 as compared to the corresponding control conditions.

First, the mean expression of genes encoding protein kinases that play a crucial role in cancer pathophysiology, namely *PI3K* (Figure 6A) and *AMPK* (Figure 6B), was determined via RT-qPCR analysis. The results showed that only the expression of gene encoding AMPK was downregulated in HPMSCs after they were exposed to 100 µg/mL of Fe_3_O_4_@Au NPs for 24 h. Next, the expression of two genes encoding protumorigenic growth factors, *PDGF* (Figure 6C) and *VEGF* (Figure 6D), was evaluated. In terms of the expression of genes encoding both PDGF and VEGF, neither was modulated over time in HPMSCs exposed to 100 µg/mL of Fe_3_O_4_@Au NPs. However, the secreted level of VEGF quantified through an ELISA after 24 h of exposure to Fe_3_O_4_@Au NPs was significantly increased compared to that under the control conditions (Figure 6E).

## 4. Discussion

Among the current therapies used to treat cancers, RT is utilized in 50% of cases for curative or palliative treatments. However, the efficiency of RT may vary considering the nature of treated tissues, as well as cancer radiosensitivity. Finding new therapeutic agents to improve, amplify, and increase the accuracy of RT appears to be crucial in the fight against cancer.

Nanotechnology has been of increased interest in the area of oncology, and identifying new radiosensitizers that can couple with ionizing radiation could bring therapeutic advances in RT. Indeed, metallic NPs seem particularly relevant in developing theranostic approaches in oncology due to their use as therapeutic and/or medical imaging agents for diagnostics [37,38,39]. Nevertheless, we still need more data about ways to transport and deliver NP into tumors, especially for cancers of deep organs.

The dual role of MSCs in cancer has been evaluated due to their immunosuppressive capacity, which promotes cancer progression, and their use as therapeutic tools in diverse pathologies [28,40,41]. Mesenchymal stem cells are particularly relevant as therapeutic vehicles, considering their recruitment at the tumor site [27,42,43]. Data from the literature reported MSCs’ tropism for inflammatory sites such as the tumor microenvironment and their accumulation in lungs, displaying a particular interest in NSCLC [29,30]. Moreover, Kidd et al. monitored, via bioluminescence, the in vivo dispersion of modified MSCs to express luciferase in syngeneic and xenogeneic breast carcinoma-bearing mice, as well as in an ovarian tumor model [31]. MSCs are recruited at the tumor site in response to various chemokines, such as CCL2 or CXCL12, secreted by CD133+ tumor stem cells, as demonstrated in mice bearing glioblastomas [34].

Engineered MSCs transport various cytokines, growth factors, inhibitors, drugs, or NPs to the tumor site for imaging or therapy [44,45,46,47,48]. Phase I clinical trials conducted in ovarian cancer and patients with advanced tumors demonstrated the feasibility of using patient-derived MSCs as carriers for therapeutic agents such as oncolytic viruses (oncolytic measles virus and oncolytic adenovirus) [44,49]. The authors demonstrated a safe combination between MSCs and oncolytic viruses and want to continue into Phase II. To consider MSCs as potential vectors for therapeutic agents such as radiosensitizers, this present study aimed to determine the impact of Fe_3_O_4_@Au NPs on the oxidative, inflammatory, and tumorigenic profiles of HPMSCs and ensure that interactions between NPs and HPMSCs would not promote cancer progression.

The core–shell NPs used in this study were produced following green chemistry principles and are constituted by a gold shell and iron oxide core [19]. Interestingly, the core–shell Fe_3_O_4_@Au NPs have unique physicochemical properties associated with decreased toxicity and enhanced biocompatibility, stability, catalytic activity, and surface functionality due to the gold shell [19,50,51,52,53]. Our previous work characterized Fe_3_O_4_@Au NPs, demonstrating them to be monodispersed and round-shaped with an average size of 8 ± 1 nm, a hydrodynamic diameter of 291 ± 40 nm, and a homogeneous distribution of gold [19].

Data showed the cellular damage induced by Fe_3_O_4_@Au NPs combined with RT in an NSCLC cellular model through apoptosis and necrosis caused by strong oxidative stress associated with an enhanced proinflammatory response.

Once the radiosensitizing effect of NPs was demonstrated, we focused on how to deliver these radiosensitizing NPs to the tumor sites and the impact of these NPs on their potential vectors. Firstly, the present study evaluated the dose and time effects of NPs on mitochondrial metabolic activities and cell viability (Figure 1). Interestingly, data from the literature show that magnetite NP-Fe_3_O_4_ exhibited a toxic effect on A549 cells from 72 h at 100 µg/mL by necrosis and apoptosis processes [54]. Otherwise, silver NPs also exhibited toxic effects through apoptosis and necrosis attributable to ROS-overproduction-mediated DNA damage on MSCs [55,56]. Comparable observations were made about zinc oxide NPs and copper oxide NPs in mesenchymal stem cells [57,58]. In contrast, our previous work on core–shell Fe_3_O_4_@Au NPs reported that these hybrid NPs did not alter A549 viability after 72 h of exposure [19]. Indeed, we demonstrated that a gold coating significantly decreases the aggregation of Fe_3_O_4_ NPs, improving stability and biocompatibility. Accordingly, results obtained on core–shell Fe_3_O_4_@Au NPs in the present study did not show the induction of cell death after 72 h of exposure or the promotion of necrosis or apoptosis at 100 µg/mL of NPs on MSCs (Figure 3).

The transport of metallic NPs needs to be non-toxic for the cells used as vectors until they reach the tumor sites. Bulk gold is known to be an inert metal relevant for medical purposes, and gold NPs do not induce adverse or acute toxicity [59,60].

The absence of toxicity of core–shell Fe_3_O_4_/Au NPs on NIH-3T3 cells suggests that these NPs are well tolerated by normal cells [61].

Our results demonstrated that MSCs internalized Fe_3_O_4_@Au NPs (Figure 2). Abdollahi et al. showed a time-dependent intracellular uptake of Fe_3_O_4_/Au NPs by labeling NPs with rhodamine on a breast cancer cell line, whereas iron oxide NPs cross the cell membrane or into vesicles by diffusion, without modifying membrane integrity [21,62,63]. Interestingly, data from the literature show that lysosomes mediated by clathrins endocytose a significant part of Fe_3_O_4_@Au NPs, and some Fe_3_O_4_@Au NPs enter the cytoplasm of HeLa cells after 3 h of incubation with NPs under or not under a magnetic field [64]. Greulich et al. demonstrated that silver NPs were taken up by cells and agglomerated inside the perinuclear region associated with the endolysosomal cell compartment of MSCs [65]. The authors concluded that the invasion route was comparable to that of other metallic NPs.

In the present study, the evaluation of the redox status showed a downregulation of the gene expression of the ROS-producing enzyme *Nox4* and no modulation of the redox transcription factor *Nrf2* over time (Figure 4). However, the results also demonstrated increased ROS production induced by Fe_3_O_4_@Au NPs and an overexpression of the gene encoding HMOX-1, suggesting oxidative stress. Accordingly, Watanabe et al. observed an upregulation of the gene encoding HMOX-1 due to the oxidative stress generated by Fe_3_O_4_ NPs at 100 µg/mL after 12 and 72 h of exposure to NPs in A549 cells [54].

The antioxidant system is a defense process activated in response to an imbalance between antioxidant and pro-oxidant species in favor of pro-oxidant species. The main enzymes constituting the antioxidant defense system are superoxide dismutases (SODs), catalase, and glutathione peroxidase (GPx), which are activated to restore redox homeostasis [66]. The ROS production induced by metallic NPs, such as titanium dioxide NPs in cultured BEAS-2B cells or Fe_3_O_4_ NPs in A549 cells, caused a dose-dependent decrease in the GSH level [54,67].

Metallic NPs such as silver NPs, gold NPs, zinc oxide NPs, and iron oxide NPs, due to their physicochemical properties, increase ROS production and decrease mitochondrial membrane potential [57,68,69]. Notably, the oxidative stress generated after exposure to metallic NPs reported in numerous studies is associated with apoptosis, necrosis, or autophagy through the modulation of the mTOR/AMPK/ERK/PI3K/Akt pathways [55,56,57,58,70,71]. In our study, an elevation in ROS production was observed in MSCs. Still, it did not induce cell viability modulation or apoptosis/necrosis activation, underscoring the potential to amplify oxidative stress at the tumor level without altering the viability of cells used as vectors.

Interestingly, in the present study, we even observed a downregulation of the proapoptotic marker, namely *caspase 9*, and an upregulation of the antiapoptotic marker, *Bcl2*, but no regulation of *caspase 3* gene expression 72 h after exposure to Fe_3_O_4_@Au NPs (Figure 3). This modulation could be due to the transient regulation of the intrinsic apoptosis pathway activated in response to excess ROS generated by nanoparticles. Importantly, caspase 9 is an initiator during the apoptosis process, while caspase 3 is an executioner. Apaf-1 and caspase 9 are inactive monomers in the cytosol, and caspase 9 initiates intrinsic apoptosis when the caspase-9 CARD domain binds to the adapter protein Apaf-1 [72].

Notably, stem cells have two developmental choices: self-renewal or differentiation. The modulation of apoptosis markers allows them to maintain a balance through the upregulation of Bcl2 and the inhibition of apoptosis, which promotes repopulation [72,73].

Notably, the present study quantified apoptotic and protumorigenic markers via RT-qPCR analysis, correlating with a modulation of gene expressions that could also be supported by a protein analysis.

Moreover, NPs also exerted anti-inflammatory effects on MSCs by regulating the release of cytokines and chemokines, except for CXCL8, after 24 and 72 h of exposure (Figure 5). Numerous data in the literature showed an upregulation of the proinflammatory response through the oversecretion of TNF-α, IL-1β or IL-6, or CXCL8 in in vitro and in vivo models [74,75]. However, other studies also demonstrated that metallic NPs exhibited a regulation of immunoregulatory molecules expressed by MSCs, associated with a decrease in the release of cytokines and chemokines [76,77].

CXCL8 is a potent chemokine known to mediate the recruitment and activation of neutrophils at the inflammatory site, as in the tumor microenvironment. However, neutrophils exhibit a controversial role in cancer progression. Indeed, neutrophils are crucial in initiating and regulating the antitumor immune system, including B cells, T cells, NK cells, and dendritic cells [78]. Neutrophils, particularly tumor-associated neutrophils (TANs) with a protumorigenic phenotype (TANs 2), are immunosuppressors and protumorigenic through the regulation of the natural killer cells and T cell phenotype (PDL1-PD1). TANs 2 also regulate tumor growth via the IL-6/STAT3 axis and proangiogenic as well as metastatic markers, such as VEGF and MMP9 [78,79].

Notably, it has been demonstrated that the extracellular traps formed by neutrophils significantly enhance gold NP trapping. Once trapped, gold NPs activate neutrophils by altering the surface charge density on neutrophil membranes, inducing NETosis [80,81].

On the other hand, zinc oxide, silver, copper oxide, and titanium dioxide NPs downregulated MSC phenotypic markers, metabolic activity, and differentiation potential, as well as the production of growth factors [77], as observed in the present study, where the Fe_3_O_4_@Au NPs decreased VEGF levels secreted by MSCs (Figure 6).

The downregulation of *AMPK* gene expression accompanied this decrease in VEGF secretion at 24 h. Multiple studies demonstrated the interplay between AMPK and VEGF in endothelial or skeletal muscle cells promoting angiogenesis [82,83]. Moreover, VEGF is also known to promote MSC differentiation through upregulating growth differentiation factor 11, leading to MSC differentiation into endothelial-like cells exerting proangiogenic activities via the ERK/EIF4E axis [84].

## 5. Conclusions

**Figure 7:**
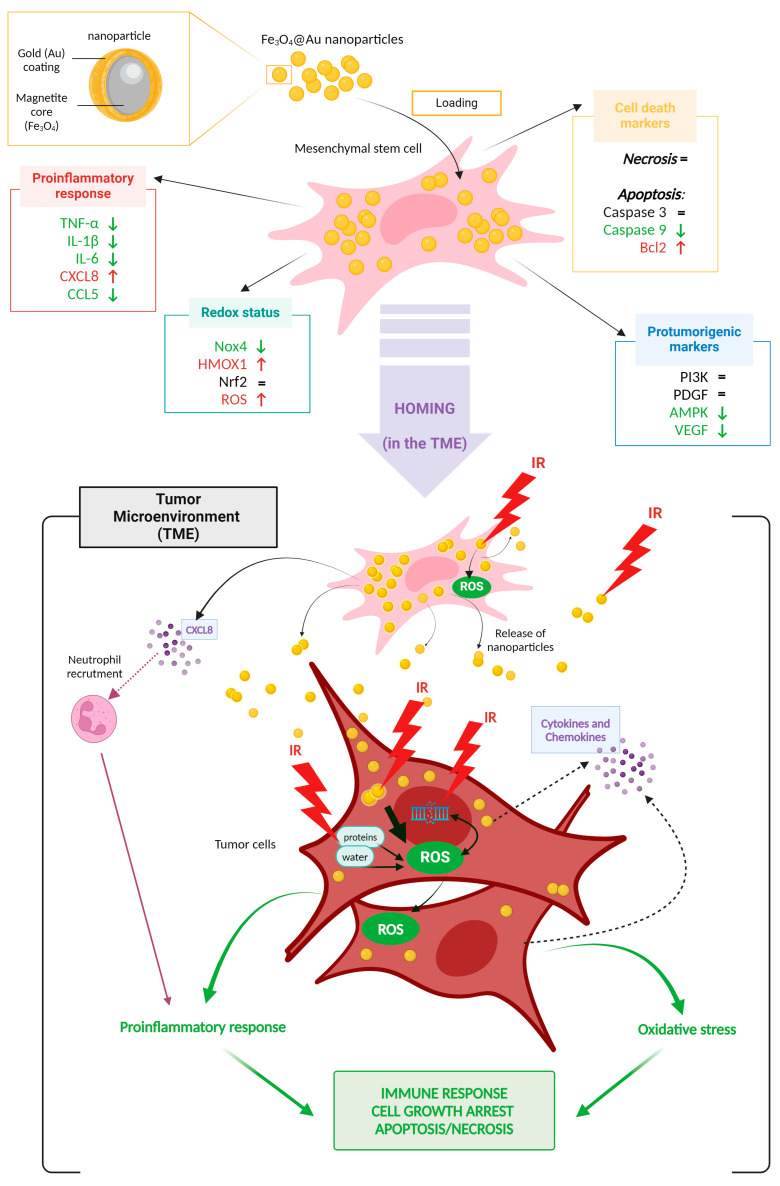
Overview of targets and activated mechanisms following nanoparticle-enriched MSC recruitment into the tumor microenvironment and combined with radiation. (Figure designed using BioRender.).

As depicted in Figure 7, mesenchymal stem cells internalize Fe_3_O_4_@Au nanoparticles without affecting cell viability or inducing necrosis. Moreover, the Fe_3_O_4_@Au nanoparticles modulate the expression and production of redox markers, suggesting oxidative stress. Moreover, nanoparticles modulate the proinflammatory response by decreasing the secreted levels of proinflammatory cytokines/chemokines, except CXCL8, which is elevated. No upregulation of the expression of genes encoding protumorigenic factors is reported, and nanoparticles even decrease the secretion of a proangiogenic marker.

These nanoparticles align with environmental concerns and represent an innovative therapeutic tool with which to enhance existing therapies. Our previous work on Fe_3_O_4_@Au NPs demonstrated the radiosensitizing effect of Fe_3_O_4_@Au nanoparticles on lung carcinoma cells [19]. Indeed, the NPs were able to potentiate the dysregulation of redox and inflammatory status caused by ionizing radiation and promote tumor cell death.

Given these results, mesenchymal stem cells could serve as promising vectors for delivering Fe_3_O_4_@Au nanoparticles to tumor sites, where they could exert their radiosensitizing effects combined with ionizing radiation, boosting the immune response, and inducing tumor cell death through increased oxidative stress, all without escalating the administered dose.

Importantly, MSCs’ dual role in carcinogenesis remains, and the risk associated with them as therapeutic carriers has to be considered with a system for controlling proliferation and a suicide system to eliminate them after treatment and ensure the safety of therapy or by providing their simultaneous death with the tumor cells via RT. This work represents a first step in evaluating the risk/benefit balance of considering MSCs as vectors in non-small-cell lung cancer (NSCLC).

## Figures and Tables

**Table 1:** Primers used to determine the expression of genes coding for markers of interest.

	Target Gene	Forward	Reverse
Apoptotic markers	*Bcl2*	TTCCTGCATCTCATGCCAAG	CTGGGAGGAGAAGATGCCC
	*Caspase 3*	TGGAACCAAAGATCATACATGGAA	TTCCCTGAGGTTTGCTGCAT
	*Caspase 9*	TGCGTGGTGGTCATTCTCTC	ATGGTCTTTCTGCTCCCCAC
Redox markers	*HMOX-1*	AAGACTGCGTTCCTGCTCAA	GGGGGCAGAATCTTGCACT
	*Nox4*	TCGCCAACGAAGGGGTTAAA	GACACAATCTAGCCCCAACA
	*Nrf2*	GCTATGGAGACACACTACTTGG	CCAGGACTTCAGGCAATTCT
Protumorigenic markers	*AMPK*	TGTCACAGGCATATGGTGGTC	GGGCCTGCATACAATCTTCC
	*PDGF*	TCCTGTCTCTCTGCTGCTAC	ATCAAAGGAGCGGATCGAGT
	*PI3K*	TCTTTGTGCAACCTACGTGA	AGCCATTCATTCCACCTGGG
	*VEGF*	ACAACAAATGTGAATGCAGACCA	GAGGCTCCAGGGCATTAGAC
Housekeeping gene	*GAPDH*	TGCGTCGCCAGCCGAG	AGTTAAAAGCAGCCCTGGTG

AMPK: AMP-activated protein kinase; Bcl2: B cell lymphoma type 2; GAPDH: glyceraldehyde-3-phosphate dehydrogenase; HMOX-1: heme oxygenase-1; Nox4: nicotinamide adenine dinucleotide phosphate oxidase 4; Nrf2: nuclear factor (erythroid-derived-2)-like 2; PDGF: platelet-derived growth factor; PI3K: phosphoinositide 3-kinase; and VEGF: vascular endothelial growth factor.

## Data Availability

All the data and materials are included in the present manuscript.
